# Evaluation of physicians’ opioid prescribing practices, attitudes, and interactions with other healthcare professionals in optimizing opioid prescribing in Pakistan

**DOI:** 10.3389/fphar.2025.1491764

**Published:** 2025-05-27

**Authors:** Hafsa Arshad, Ali Hassan Gillani, Muhammad Arshed, Yu Fang

**Affiliations:** ^1^ Department of Pharmacy Administration and Clinical Pharmacy, School of Pharmacy Xi’an Jiaotong University, Xi’an, Shaanxi, China; ^2^ Center for Drug Safety and Policy Research, Xian Jiaotong University, Xi’an, Shaanxi, China; ^3^ Shaanxi Center for Health Reform and Development Research, Xi’an, Shaanxi, China; ^4^ University Institute of Public Health Faculty of Allied Health Sciences, The University of Lahore, Lahore, Pakistan

**Keywords:** opioid prescribing practices, opioids, optimizing opioid prescribing, Pakistan, opioid stewardship program

## Abstract

**Background:**

The community is facing an imminent opioid crisis, with prescription opioids playing a significant role. We conducted a study to assess physicians’ experience with opioid prescribing, their reported degree of confidence in prescribing, any adverse events that occurred, and their interactions with other healthcare providers regarding opioid medication.

**Methods:**

From October 2023 to March 2024, physicians in three cities in Punjab, Pakistan, participated in a physical, cross-sectional survey based on questionnaires. A 56-item questionnaire was distributed to physicians working in both public and private hospitals. The demographics and other items were evaluated using descriptive statistics, and the associations between knowledge, attitude, and concerns were tested using the Spearman rho test. The statistical analyses were conducted using SPSS.

**Results:**

Of the 1,009 physicians contacted for the study, 816 (80.9%) answered and completed the survey. The average age of these physicians was 38 years. More than half of the respondents were men, accounting for 58.8% of the total, and an equal proportion of respondents, 56.9%, were from government hospitals. Most individuals (92.2%) were knowledgeable about the possible hazards and negative consequences linked to opioid therapy, and almost as many were aware of the risk factors associated with opioid misuse (90.2%). The majority of physicians displayed a favorable attitude, with 54.9% expressing confidence in their ability to detect patients with a strong propensity for addiction. The majority of physicians expressed worries regarding the use and management of opioids, with only 11.8% indicating no concern about legislation and 7.8% indicating no concern about overdose and abuse. Half (51.0%) of the participants never prescribed opioids, while 5.9% had a tendency to regularly prescribe them to patients. There is a strong positive linear correlation between knowledge and attitude, knowledge and concerns, and attitude and concerns.

**Conclusion:**

Prescribers were well aware of risk factors and abuse-related problems and showed a good attitude and positive concerns regarding opioid use. The practices are moderately appropriate and can be further refined by incorporating the proper guidelines and legislations in the curricula.

## 1 Introduction

Prescribing opioids in pain, either malignant or non-malignant, is among the top challenges in the healthcare system (Mills et al., 2019; International Pain Summit of the International Association for the Study of Pain, 2011). Both non-opioid and opioid analgesics are used in the treatment of pain, but opioids carry a high likelihood of dependence and addiction (Majid et al., 2019). This addiction is related to unsafe and irrational use of opioids that leads to an increased burden on the healthcare system. The results of one systematic review revealed that 36% of non-cancerous patients used opioids unsafely, whereas another previous review stated that 76% of patients misused opioids (Jantarada et al., 2021; Vowles et al., 2015). Opioid addiction may lead to death due to overdose, and by 2009, deaths by prescription drugs, including prescription opioids, exceeded the casualties due to roadside accidents (Paulozzi, 2012; Mack et al., 2013). Every day, almost 115 people die in the United States of America (USA), and in 2023, more than 79,000 US citizens died as a result of any opioid overdose, including prescription opioids, heroin, and fentanyl-like agents (Majid et al., 2019; CDC, 2025). Due to a surge in the use of opioids and the insufficiency of laws governing different states to alleviate the use, the first Trump administration, on 26 October 2017, declared the opioid crisis a national health emergency. This emergency requires an overhaul of the basic response to this problem, that is, managing opioid prescriptions and decreasing opioid use disorders (Rutkow and Vernick, 2017).

Pakistan is currently in the top 10 countries with the highest incidence of opioid addiction. The World Drug Report of 2022 reported that 284 million individuals were involved in both prescription and nonprescription opioid abuse in 2022 (Iqbal et al., 2023; Rahool, 2023). Many factors may lead to an increase in fatalities due to prescribed opioid poisoning. However, physicians hold the cornerstone position to avoid such a catastrophe as they encounter chronic pain patients who are managed by opioid medications in outpatient wards (Daubresse et al., 2013; Caudill-Slosberg et al., 2004). Most physicians are indifferent to practice recommended strategies while prescribing opioid prescriptions, that is, inadequate rating by pain scale, misunderstanding of the fears of prescribing fentanyl-like agents to opioid-naïve patients (opioid-naïve” means a patient who has not used opioids for more than seven consecutive days during the previous 30 days), hindered use of presumptive toxicology screens on patients either before or in-between opioid therapy, or resistance to discontinue opioids if patients fail to meet the treatment goal (Majid et al., 2019; Tournebize et al., 2016). Although there is a lack of up-to-date data on prescription opioid usage in Pakistan, a 2018 study reported that there was a drastic increase in tramadol prescriptions from 2004 to 2015 in developing nations, including Pakistan (Iqbal et al., 2023; Vijayan et al., 2018). It was also reported that Pakistani physicians have a high preference to prescribe tramadol to treat chronic pain in the country (Vijayan et al., 2018; Iqbal et al., 2021). Opioids such as nalbuphine, tramadol, and codeine remain easily accessible over the counter in licensed pharmacies and medicine retail outlets without a prescription (Bashir et al., 2021).

Opioid stewardship follows the same principle as antibiotic stewardship: “Right medication use for right patient at right time” (Uritsky et al., 2021). Stewardship offers the principle of rationalizing the medication, and it can be achieved by improving prescribing practices, tailoring prescribers’ knowledge and behaviors, and incorporating shared decision-making in the healthcare system (Shrestha et al., 2023). Numerous policies and suggestions have been made to manage opioid use, but most of them are ineffective and overlook basic principles of rational prescribing (Shrestha et al., 2023). With the mounting trends of opioid abuse, it is imperative to study the prescribing practices among physicians, their attitudes, and beliefs toward this issue. It is also obligatory to highlight the provider–patient and provider–healthcare interaction regarding the possible risks of opioids and appraise alternative therapies. If prescribing opioids seems pertinent, they should be administered as a function of the extensive trauma.

We aimed to conduct a study in Punjab evaluating the prescribing practices, knowledge, and attitudes toward opioids and the stewardship program. We also captured the prescribers’ ease and concerns while recommending opioids, and their interactions with nurses and pharmacists.

## 2 Methodology

### 2.1 Study area and design

Pakistan comprises four provincial localities, Punjab, Baluchistan, Sindh, and Khyber Pakhtunkhwa, and it is also a land of two independent administrative territories, Gilgit Baltistan and Azad Jammu Kashmir. Provinces are subdivided into divisions, districts, tehsils (administrative areas that consist of towns), and towns. Though the land occupied by Punjab is only 26% of Pakistan's total area, it is the most populous of all provinces, home to 60% of the country’s population (Pakistan Bureau of Statistics, 2021). The government offers healthcare through a three-tiered healthcare delivery system, which provides coverage for approximately 10% of the population. Basic health units and rural health centers provide the foundation of primary healthcare. Secondary care is delivered by tehsil and district headquarters, which encompass both first and second-referral facilities, as well as tertiary care supplied by teaching institutes. The private health sector incorporates an extensive array of medical professionals, including physicians, nurses, pharmacists, traditional healers, and laboratory technicians (Wikipedia, 2023). The physician-to-patient ratio in Pakistan is 1.1 for 1,000 patients, which is much lower than the standard values, and most of them are engaged in private practices (Economics, 2023).

This multisite study was performed using the cross-sectional study design. We have gathered information from the physicians working in government hospitals and private hospitals. These locations were selected through convenience sampling, but they carry a limitation in that such sampling lacks proportionate representation of the entire population, resulting in lower generalization of research findings than probability sampling.

### 2.2 Survey population and survey administration

Our target population was practicing physicians in the government and private hospitals from the three cities (Lahore, Sahiwal, and Bahawalpur) of the Punjab, Pakistan. Data were collected by three teams of data collectors (mostly students) who were trained to collect the data according to the protocol. These hospitals provided both inpatient and outpatient services for the population. We targeted the physicians providing services in inpatient wards and outdoor clinics. The included specialties were general practitioners, obstetricians and gynecologists, oncologists, surgeons, dentists, and orthopedists. Physicians working in academia, on leave, or holding administrative posts in hospitals were not included.

The study was conducted from October 2023 to April 2024. Initially, we approached the potentially eligible physicians in hospitals to ascertain their willingness to participate in the study. We extended invitations to nearly all the accessible physicians during the time of the visit, and those who expressed their willingness and availability were provided with the questionnaire to complete on the spot. The primary issues encountered were a high patient load and limited time availability of the doctor. To address this challenge, we approached the physicians during their break and provided them with the questionnaire. We subsequently gathered the completed forms after some time. Even after this, if we faced a lack of success, we approached the physicians as patients and politely asked if they would be willing to provide the data. Physicians who remained unwilling to participate were excluded. Prior to the initiation of data collection, a cover letter was provided to participants who agreed to participate. The cover letter communicated to physicians the need to maintain anonymity in their responses, emphasized the voluntary nature of their involvement, and provided physicians with an understanding of the objective of our study. Physicians were asked to sign the letter indicating that they approved of the participation. We asked the participants to complete the questionnaire on the spot, but if they were not free to complete it, they could return the completed questionnaire via mail or deliver it to a team member visiting to collect. We assured the participants that this survey was for research purposes and that the confidentiality of their responses would be maintained to ensure the appropriateness of the data. We also explained that the study was conducted by students of Xi’an Jiaotong University, China, to improve the standards of opioid prescribing, strengthening the policies to rationalize the use and aligning the healthcare professionals’ role in managing the opioid crisis in Pakistan. A questionnaire was considered useful if it was filled out and provided a single answer for questions requiring one response, and so on. To carry out the thorough examination of data completeness, the principal investigator independently verified and entered the data in MS Excel, which was cross-checked by AHG.

### 2.3 Tool development and validation

A self-administered, structured questionnaire comprising five sections and 64 response items that spanned four pages was distributed to respondents in our study. This survey instrument was developed based on a study of relevant literature (Majid et al., 2019; Wenghofer et al., 2011; Morley-Forster et al., 2003; Hutchinson et al., 2007; Nwokeji et al., 2007), which we further improved through discussions between the authors (MA, HA, and YF). The fundamental use of this form is the reporting of opioid-related adverse events (e.g., by number of patients, type of opioid, and cause of the event) and physicians' concerns regarding the different effects of opioid prescribing on patients. We also measured physicians’ comfort, confidence, and satisfaction with opioid prescribing; their views on approaches to adjust their prescribing; and their interactions with other healthcare professionals.

The first section of the questionnaire had information regarding gender, years of practice, age, medical specialty, practice location, and type of hospital. The second component consisted of 11 questions pertaining to the understanding of opioids, opioid stewardship, and the resolution of opioid-related issues. The responses were recorded in binary format (yes/no), and by selecting the appropriate answer, the correct response was collected. Section 3 of the questionnaire consisted of seven questions that examined attitudes and 15 questions that examined prescribing practices related to opioids. The responses were collected using a 5-point Likert scale for prescribing attitudes. This section included an additional 15 questions about opioid prescribing practices. These questions inquired about the number of patients who were administered opioids, the reasons for not prescribing opioids if none were provided, the reporting of adverse events related to opioid usage, and the factors of opioid adverse events. The questions were based on attitudinal queries frequently utilized in surveys regarding physician attitudes and practices (Hutchinson et al., 2007; Nwokeji et al., 2007). Explorations suggest that these beliefs are linked to physicians’ tendency to prescribe opioids (Wenghofer et al., 2011; Morley-Forster et al., 2003; Hutchinson et al., 2007; Nwokeji et al., 2007). Seven items were present in Section 4 of the questionnaire, which asked about physicians' concerns when prescribing opioids, and a 4-point scale evaluated the section (not at all concerned, a little concerned, somewhat concerned, and very concerned). The last section included information on the interaction of physicians with other health care providers (HCPs) (17 items: frequently, sometimes, and not at all). The questionnaire is available as Supplementary Material S1.

Before commencing the research, the tool was piloted with 11 general medicine physicians, six gynecologists, and six orthopedists to evaluate the lucidity and comprehensibility of the tool items. Pre-testing also helped us determine whether respondents were willing to participate in the study and provide the necessary information. Twenty-three physicians participated in the pretest survey, and their responses were not included in the original data. Based on their feedback, minor alterations were made to clarify the wording and scaling.

The construct and content of the questionnaire were validated by an extensive literature review, ensuring that the data collection instrument accurately measured the intended hypothesis. Professional researchers examined the items in the data collection instrument and provided their professional comments to guarantee face validity. The designed questionnaire underwent a review and evaluation by four professors with a background in pharmacy. Their aim was to analyze the suitability of each item for measurement purposes. The instrument was revised based on the expert comments obtained through discussions with researchers. Internal consistency of the questionnaire was measured by Cronbach’s alpha. Cronbach’s alpha of the questionnaire was 0.669≈0.70, which shows that the reliability of the research tool is acceptable.

### 2.4 Statistical analysis

An analysis of descriptive statistics was conducted to examine the demographics and other variables. Percentages were used to represent the ordinal data, while the mean and standard deviation were used for continuous variables. The responses to each item in the knowledge, attitude, and concerns section were recorded and are represented as numerical values and percentages. We employed Spearman’s rho correlation test to assess the relationship between the knowledge, attitude, and concern sections. The significance threshold was set at 0.001. To assess the correlation, we transformed the knowledge (items 1–11) into scores by assigning a value of 1 to the correct answer and 0 to the incorrect response. In order to obtain the scores for the attitude portion (items 3–9), we aggregated the responses, assigning a value of 5 to “strongly agree,” 4 to “agree,” 3 to “neutral,” 2 to “Disagree,” and 1 to “Strongly disagree.” Similarly, the concern section (items 2–9) was transformed into scores by assigning a numerical value to each response. For instance, a response indicating “very concerned” was assigned a score of 4, “somewhat concerned” was assigned a score of 3, “a little concern” was assigned a score of 2, and “no concern at all” was assigned a score of 1. The scores were aggregated and utilized in correlation.

### 2.5 Ethical approval

The study received ethical approval from the Bioethics Committee of Xi’an Jiaotong University, China (2023-21-PA). Prior to beginning, individual physicians were mandated to submit written consents. The researchers abstained from soliciting any personal information from the individuals. In addition, every participant received an assurance that their data would exclusively be utilized for research purposes and would remain anonymous.

## 3 Results

### 3.1 Demographics

Of the 1,009 physicians approached for the study, 816 (80.9%) responded to the study and completed the questionnaire. Their age ranged from a minimum of 25 to a maximum of 55 years, with a mean age of 38 years. The practice years ranged between 1 year and 30 years, and the mean was 16 years. More men (58.8%) participated than women (41.2%), and similar percentages of respondents worked at government hospitals (56.9%) vs. private settings (43.1%). The largest group of participants was general practitioners (47.1%), followed by surgeons (19.6%). All the data are available in Table 1.

**TABLE 1:** Demographic information of physicians.

	Number	Percentages
Gender		
Men	480	58.8
Women	336	41.2
Practice setting		
Government	464	56.9
Private	352	43.1
Age		
25–35 years	224	27.5
36–45 years	560	68.6
46–55 years	32	3.9
Nature of hospital care services		
Primary care hospital	96	11.8
Secondary care hospital	272	33.3
Tertiary care hospital	448	54.9
Medical specialty		
General practice	384	47.1
Obstetrics and Gynecology	112	13.7
Surgery	160	19.6
Others (Dental, Orthopedics)	160	19.6

### 3.2 Knowledge about opioids and opioid stewardship

Regarding knowledge about opioids and opioid stewardship, respondents are highly aware of most of the questions. The “yes” responses vary from a minimum of 51.0% (indicating awareness that opioids are classified under the G schedule of the drug regulatory authority of Pakistan) to a maximum of 96.1% (indicating knowledge of opioid drugs). Remarkably, the participants exhibited a high level of awareness regarding the potential hazards and negative consequences linked to opioid therapy (92.2%) as well as the factors that increase the likelihood of opioid abuse and addiction (90.2%). When rating the opioid items, the maximum score is 11. In our study, the participants were aware of a maximum of 11 questions related to opioids, while the minimum number of questions answered was 2. The mean (standard deviation) of the score was 8.14 (2.37), indicating that individuals had a good understanding of opioids (Table 2).

**TABLE 2:** Knowledge about opioids and opioid stewardship.

No.	Questions	Responses			
		Yes		No	
		N	%	N	%
1	Do you know about opioid stewardship? (Opioid stewardship refers to a series of strategies and interventions involving the appropriate procurement, storage, prescribing, and use of opioids, as well as the disposal of unused opioids when opioids are appropriately prescribed for the treatment and management of specific medical conditions.)	544	66.7	272	33.3
2	Do you know about opioid drugs?	784	96.1	32	3.9
3	Do you know about the WHO analgesic ladder of pain management?	640	78.4	176	21.6
4	Do you know about the CDC guideline for prescribing opioids for chronic pain?	464	56.9	352	43.1
5	Are you aware of the potential risks and adverse effects associated with opioid therapy?	752	92.2	64	7.8
6	Do you know opioid drugs are included in Schedule G drugs by the Drug Regulatory Authority of Pakistan	416	51.0	400	49.0
7	Do you know opioid stewardship programs can help reduce the risk of opioid addiction and abuse?	592	72.5	224	27.5
8	Do you know about guidelines and recommendations for prescribing opioids in chronic pain management?	576	70.6	240	29.4
9	Do you have adequate knowledge of the appropriate use of naloxone in opioid overdose situations?	576	70.6	240	29.4
10	Are you familiar with the risk factors for opioid abuse and addiction?	736	90.2	80	9.8
11	Do you aware of the resources available for patients who require assistance with opioid tapering or addiction treatment?	560	68.6	256	31.4

### 3.3 Physicians’ practices, attitudes, and confidence about opioid prescribing

The percentage of individuals who expressed positive attitudes and confidence toward opioid prescribing for chronic pain increased significantly from 5.9% to 64.7% when considering concerns about potential opioid diversion or abuse in their practice setting. A notable finding was that a mere 19.6% of participants, or 1 in 5, reported feeling satisfied when prescribing opioids for pain patients in their respective settings (the details are presented in Table 3). The items were rated on a 5-point Likert scale, with a rating of 5 indicating strong agreement and a rating of 1 indicating severe disagreement. The score spans from 7 to 35. Of all the participants, 16 individuals (2% of the total) had the lowest attitude and scored 7. On the other hand, 32 respondents (3.9% of the total) achieved the highest score of 29.

**TABLE 3:** Attitude and confidence of physicians about opioid prescribing.

No.	Questions	Strongly agree		Agree		Neutral		Disagree		Strongly disagree	
		N	%	N	%	N	%	N	%	N	%
1	I am comfortable prescribing opioids for chronic pain	0	0	48	5.9	352	43.1	240	29.4	176	21.6
2	I am confident in my clinical skills in prescribing opioids	32	3.9	192	23.5	336	41.2	176	21.6	80	9.8
3	In my practice, many pain patients experience substantial pain relief with opioids	112	13.7	304	37.3	224	27.5	96	11.8	80	9.8
4	I find it satisfying to prescribe opioids to pain patients	64	7.8	96	11.8	224	27.5	240	29.4	192	23.5
5	I am confident about incorporating non-opioid pain management strategies into my practice, such as physical therapy or cognitive–behavioral therapy	176	21.6	208	25.5	240	29.4	112	13.7	80	9.8
6	I am concerned about the potential for opioid diversion or abuse in my practice setting	256	31.4	272	33.3	192	23.5	48	5.9	48	5.9
7	I am confident in my ability to identify patients who may be at high risk for opioid abuse or addiction	160	19.6	288	35.3	224	27.5	64	7.8	80	9.8

### 3.4 Physicians’ opioid prescribing practices

**FIGURE 1:**
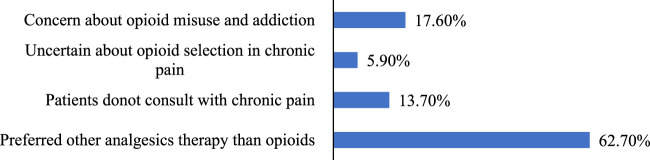
Why physicians do not prescribe opioids in their practice.

**FIGURE 2:**
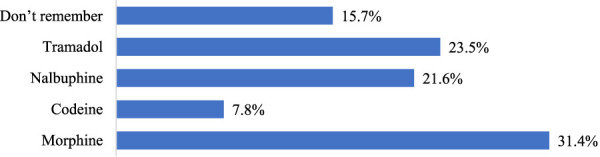
Opioid involved in the most recent adverse event in the physician’s practice.

Upon examining the prescribing practices, we discovered that over half (51.0%) of the physicians never prescribed opioids in their practice, whereas only 5.9% prescribed opioids to more than 200 patients, 32 (3.9%) physicians prescribed opioids to 1–100 patients, and 32 (3.9%) physicians prescribed opioids to 100–200 patients in past 3 months. Figure 1 illustrates the factors that contribute to the decision not to prescribe opioids. Of the entire sample, 31.3% of the physicians had experienced an adverse opioid drug event in their practice in the past 3 months. Figure 2 illustrates the predominant opioids responsible for the occurrence of adverse events.

**FIGURE 3:**
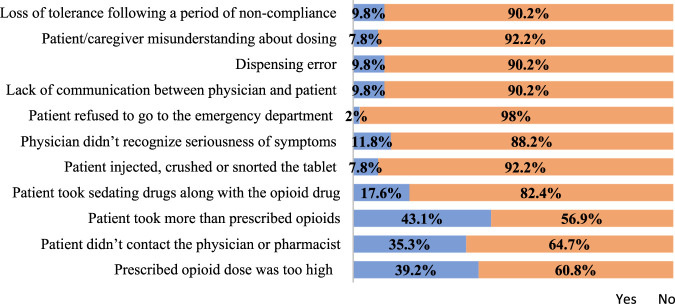
Factors contributing to adverse events related to opioids in the physician’s practice.

Physicians reported morphine was involved in 31.4% of adverse events during their prescribing practices. Participants also highlighted contributing factors of adverse events due to opioids in their practices: 39.2% of physicians reported that the prescribed opioid dose was too high during the occurrence of adverse events, whereas 43.1% of adverse events were reported because the patient took more opioids than were prescribed. All other factors responsible for adverse events due to opioids are listed below in Figure 3.

### 3.5 Extent of physician concerns while prescribing opioids

Nearly one in five (19.6%) of the prescribers expressed indifference to patients' drug addiction and non-compliance with therapy. Likewise, a minor fraction (11.8%) had no concerns regarding issues with legislation and expectations from patients. The specific information is provided in Table 4.

**TABLE 4:** Physician’s concerns while prescribing opioids.

Extent of physician concerns while prescribing opioids									
No	Prescribing concerns	Not at all concerned		A little concerned		Somewhat concerned		Very concerned	
		N	%	N	%	N	%	N	%
2	Patient’s addiction to opioids	160	19.6	160	19.6	192	23.5	304	37.3
3	Non-compliance to therapy (e.g., missed appointments)	160	19.6	144	17.6	272	33.3	240	29.4
4	Trouble with legislation	96	11.8	256	31.4	224	27.5	240	29.4
5	Demand for early refill, unrealistic expectations of pain, demanding fit-in appointments, and lost prescriptions	96	11.8	192	23.5	304	37.4	224	27.5
6	Overdose and abuse	64	7.8	128	15.7	240	29.4	384	47.1
7	Lack of resources to treat addiction	112	13.7	80	9.8	256	31.4	368	45.1
8	Patient’s unwillingness about opioid uses	128	15.7	176	21.6	368	45.1	144	17.6
9	Lack of specialized pain clinics	128	15.7	144	17.6	320	39.2	224	27.5

### 3.6 Physician interactions with other healthcare providers (pharmacists, nurses) regarding opioid therapy (pharmacist and nurse)

Fewer than half (41.2%) of the physicians surveyed reported that they had never engaged with pharmacists regarding opioid therapy and prescriptions. Conversely, only 21.6% indicated that they had never engaged in any form of communication with nurses regarding opioid therapy. The primary issue found when contacting a pharmacist is that the pharmacist appeared to lack expertise on opioids, with a prevalence of 69.6%. This was followed by difficulty reaching directly due to communication gaps, accounting for 66.7% of the cases (Table 5). Most respondents (64.7%) reported that pharmacists effectively addressed their inquiries or concerns regarding opioids. Most respondents had a positive perception of nurse-physician interactions. Specifically, 76.5% agreed that nurses accurately assessed opioid intoxication, withdrawal, or pain. Additionally, 70.6% of respondents reported that nurses occasionally or frequently encouraged them to prescribe benzodiazepines or analgesics to keep patients calm or quiet (Table 5).

**TABLE 5:** Interaction of physicians with other healthcare providers regarding opioid therapy (pharmacist and nurse).

1	How often do you interact with pharmacists regarding opioid therapy/prescriptions?						
	Weekly 160 (19.6%) Monthly 208 (25.5%) Quarterly 112 (13.7%) Never 336 (41.2%)						

No.	Situations encountered with a pharmacist regarding opioid therapy	Frequently		Sometimes		Not at all	
		N	%	N	%	N	%
2	Pharmacist was difficult to reach directly due to a communication gap	224	27.5	320	39.2	272	33.3
3	Pharmacist called to verify something that is already stated on the Rx	112	13.7	352	43.1	352	43.1
4	Pharmacist requested a change in the wording of opioid prescriptions	112	13.7	352	43.1	352	43.1
5	Pharmacist challenged a prescription that I felt was appropriate	112	13.7	320	39.2	384	47.1
6	Pharmacist made a recommendation to a patient that I felt was inappropriate	160	19.6	304	37.3	352	43.1
7	Pharmacist interacted for minor issues or non-emergencies in opioid therapy management	160	19.6	272	33.3	384	47.1
8	Pharmacist dispensed opioid earlier than the time stated on the Rx, e.g., on a Rx dispensed weekly	128	15.7	304	37.3	384	47.1
9	Pharmacist did not seem very knowledgeable about opioids	176	21.6	400	49.0	240	29.4
10	Pharmacist dispensed opioid medication without Rx when they could not reach the physician	128	15.7	368	45.1	320	39.2
11	Pharmacist did not adequately answer my concerns about opioids	176	21.6	352	43.1	288	35.3
12	How often do you interact with a nurse regarding opioid therapy/prescriptions?						
	Weekly 160 (19.6%) Monthly 240 (29.4%) Quarterly 240 (29.4%) Never 176 (21.6%)						

	Situations encountered with a nurse regarding opioid therapy	Frequently		Sometimes		Not at all	
		N	%	N	%	N	%
13	Nurse felt uncomfortable while administering the opioids that I prescribed	112	13.7	400	49.0	304	37.3
14	Nurse showed reluctance with my opioid prescription to inpatients	96	11.8	368	45.1	352	43.1
15	Nurse felt that the patient was drug seeking or difficult, and I did not necessarily agree	64	7.8	496	60.8	256	31.4
16	Nurse pressured me to prescribe something to keep the patient calm or quiet, e.g., benzodiazepines or other pain killers	144	17.6	432	52.9	240	29.4
17	Did not feel that the nurse’s assessment of opioid intoxication, withdrawal, or pain was accurate	144	17.6	480	58.8	192	23.5

### 3.7 Demographic associations with knowledge, prescribing attitude, and concerns score

Table 6 presents the results of demographic associations of the respondents with the knowledge, attitude, and concerns scores. Analysis revealed that men had statistically high knowledge and practice scores, whereas women had higher concern scores. Respondents working at private hospitals scored statistically significantly higher than respondents working at government hospitals (P < 0.001).

**TABLE 6:** Demographic association with knowledge, prescribing attitude, and concerns score.

No.	Demographics	Knowledge score		Prescribing attitude		Concerns score	
		Mean (SD)	P value	Mean (SD)	P value	Mean (SD)	P value
1	Gender						
	Men	8.62 (1.86)		22.33 (4.94)		21.67 (6.50)	
	Women	7.80 (2.62)	<0.001	21.23 (4.39)	0.001	23.47 (5.62)	<0.001
2	Practice setting						
	Government	7.93 (2.54)		20.90 (5.23)		21.67 (5.53)	
	Private	8.41 (2.14)	0.004	22.73 (3.51)	<0.001	24.00 (6.48)	<0.001
3	Age						
	25–35 years	8.57 (1.88)		20.21 (5.80)		20.07 (6.04)	
	36–45 years	7.86 (2.53)		22.06 (4.02)		23.57 (5.85)	
	46–55 years	10.0 (1.01)	<0.001	25.50 (1.52)	<0.001	26.50 (2.54)	<0.001

### 3.8 Correlation between knowledge, attitude, and concerns of physicians regarding opioid use

When we examined the relationship between knowledge, attitude, and concerns, we found a substantial positive connection between the knowledge score and attitude score (r = 0.275, p = 0.01), the knowledge score and concerns score (r = 0.120, p = 0.01), and the attitude score and concerns score (r = 0.495, p = 0.01). The details are given in Table 7.

**TABLE 7:** Correlation between knowledge, attitude, and concerns scores of physicians regarding opioid use.

Test	Variables	Correlations		
		Opioid knowledge score	Attitude score	Prescribing concern score
Spearman’s rho	Opioid knowledge score	1.000		
	Attitude score	0.275**	1.000	
	Prescribing concerns score	0.120**	0.495**	1.000

Correlation is significant at the 0.01 level (2-tailed).

## 4 Discussion

Medicines can be obtained without a prescription in Pakistan due to the lack of implementation of regulations and rules. As a result, patients sometimes self-medicate based on past prescriptions (Iqbal et al., 2023; Gillani et al., 2020). Factors contributing to the unsafe use of opioids include uncontrolled pain, socio-cultural influences, sharing of opioids, excessive prescribing of opioids, influence of pharmaceutical advertising, inadequate regulation, easy access to medications, preference for injectables, patient non-compliance with treatment plans, lack of regulation, untrained or unlicensed staff in medicine outlets, and financial or economic deficits (Iqbal et al., 2023; Iqbal et al., 2021; Bashir et al., 2021; Jalali et al., 2020; Gourlay et al., 2005). Our study in Pakistan aimed to estimate the following for the first time: physicians’ knowledge about opioids and opioid stewardship, physicians’ attitudes, the number of patients prescribed opioids, physicians’ concerns about opioid therapy, the number of opioid-related adverse events experienced by patients, and interactions of physicians with other HCPs to optimize opioid therapy. These figures are moderately alarming and indicate a pressing necessity for educational intervention.

Our study found that most physicians were knowledgeable about the WHO Pain Ladder management, the CDC guidelines for opioid prescribing, and the inclusion of these medications in Schedule G by the Drug Regulatory Authority of Pakistan (DRAP) (Schedule G contains the list of drugs that should be used under medical supervision only). According to a previous survey from Pakistan, most physicians prescribed opioids based on the pain scale (Iqbal et al., 2021). The pain score is a crucial component of the “universal precautions” method for evaluating the likelihood of opioid misuse in people with chronic pain (Gourlay et al., 2005). In addition, physicians were cognizant of potential side effects and strategies for managing opioid overdose and tapering. Consistent with previous findings, physicians advised their patients about drug tapering prior to prescribing opioids, with a positive response rate of 71.6% (n = 235) among participants (Majid et al., 2019). Approximately half of the group abstained from giving opioids to patients, while only 5.9% had a regular practice of prescribing them for non-chronic pain. These facts indicate that physicians were cognizant of the detrimental effects associated with opioid usage, as well as the risk factors for opioid abuse and addiction. Similar outcomes have been observed in prior research studies (Majid et al., 2019; Iqbal et al., 2021). It may also be a depiction of the Know-to-do gap in physician knowledge regarding confidence in them, skills to choose opioids, and high satisfaction in prescribing opioids. Only a minority of respondents in our findings expressed comfort in writing prescriptions, confidence in their ability to assess and treat patients with chronic pain, and satisfaction with prescribing opioids. Multiple studies, encompassing different medical specialties such as family physicians, surgical trainees, and obstetrician-gynecologists, have consistently highlighted the need for improved training and education on opioid prescribing (Roy et al., 2017; Nooromid et al., 2018; Madsen et al., 2018). Furthermore, a significant portion of our community exhibits a strong inclination toward integrating non-opioid pain management options into their professional practice. In a separate study, the intervention group was educated about evaluating the likelihood of opioid usage. As a result, there was a significant rise in the group’s enthusiasm to utilize risk assessment (Kavukcu et al., 2015). Medical schools can improve their curriculum by including content that directly addresses the escalating opioid issue. By implementing this approach, they can provide future physicians with the essential knowledge and abilities required to effectively recognize the indications, symptoms, and hazards linked to opioid consumption, ultimately resulting in enhanced patient outcomes (Ratycz et al., 2018). However, the Drug Abuse Control Master Plan 2010–2014 of Pakistan (Government of Pakistan, 2010) fails to address the management of the opioid pandemic resulting from the use of prescription opioids. Nevertheless, professionals possess the requisite foundational knowledge and utilize appropriate protocols when it comes to the administration of opioids. The manual mentioned above should offer suitable criteria to optimize the efficacy of opioid prescribing.

While acknowledging the concerns, it was noted that a significant portion of the targeted community expressed worry regarding patients' dependence on opioids, the risk of overdose and abuse, and the insufficient availability of resources to address addiction issues. Canadian physicians (Wenghofer et al., 2011) have also emphasized these problems. Additional research supports the findings and confirms that these concerns are valid (i.e., both medically prescribed and illegally obtained opioids contribute to overdoses and addiction). For instance, of 1,095 individuals enrolled in the Ontario Drug Benefit Program who died due to opioid overdose, 56% had been prescribed opioids within 4 weeks prior to their deaths (Dhalla et al., 2009). Implementing safe practices for prescription opioids will effectively reduce these dangers. Additionally, safe practices will restrict the availability of diverted opioids, as the quantity of diverted opioids is directly linked to the quantity of prescribed opioids. The acquisition of knowledge directly influences mindset, leading to a more favorable outcome. There is a significant positive linear correlation between the knowledge score, attitude scores, and concern score. Previous research has shown that a positive understanding leads to favorable results (Gillani et al., 2019).

The stewardship program is a collaborative effort, including healthcare professionals from several disciplines, typically led by a practitioner with specialized knowledge in the use of medicine, often a physician and/or pharmacist (SuHa et al., 2017). The success of the stewardship program hinges on the collaboration between the leadership, the team, and the essential support groups. The composition of team members can differ based on the institution and available resources. This may encompass clinicians, infection prevention specialists, nurses, epidemiologists, pharmacists, laboratory staff, information technology workers, and management (SuHa et al., 2017). Two-fifths of the respondents were in communication with a pharmacist, while one-fifth were in contact with nurses to assist in optimizing the opioid therapy. The values in this study are significantly lower than those reported in a previous study conducted in Canada (Wenghofer et al., 2011).

Relying primarily on a blanket approach that focuses solely on prescription numbers and doses, without considering patient-reported outcomes, is insufficient to effectively reduce the use of opioids. It is crucial to engage patients, caregivers, and a diverse group of HCPs who are involved in their treatment (Shrestha et al., 2023; Australian Commission on Safety and Quality in Health Care, 2022). A prior study established a significant correlation between patient-reported outcomes and the chance to engage with HCPs and broader opioid stewardship programs (So, 2021). Our results indicate notable demographic and institutional differences in opioid prescribing practices (Table 6). Men scored higher in both knowledge and practice than women, which may reflect cultural norms in Pakistan’s healthcare system, where male physicians are more likely to hold leadership roles and have greater access to training. In contrast, female providers exhibited higher concern scores, possibly due to a heightened awareness of opioid risks or societal pressures to exercise caution. Notably, physicians in private hospitals significantly outperformed those in government settings across all measures (p < 0.001), highlighting systemic inequities. Private practitioners likely benefit from more current resources, patient-focused incentives, and stronger interdisciplinary collaboration, whereas government providers face bureaucratic constraints, outdated training, and resource limitations that may hinder adherence to best opioid prescribing practices (Javed et al., 2019). The combined impact of gender and institutional barriers, particularly for women in government settings, exacerbates these gaps. Addressing these disparities will require gender-sensitive training programs to bridge opioid knowledge gaps, as well as systemic reforms in government hospitals, including updated guidelines, fair resource allocation, and enhanced interdisciplinary teamwork to optimize opioid prescribing practices in Pakistan.

### 4.1 Strengths and limitations

We recognize the constraints of our study. This study inquired about chronic pain, encompassing both cancer-related pain and palliative pain. It is important to note that distinct opioid prescribing rules are applicable to palliative patients. This study was limited to a cross-sectional snapshot and did not inquire about the respondents' interest in further exploring specific clinical subjects, such as the utilization of addiction screening instruments. These aforementioned concerns can be resolved through future studies that aim to confirm the dependability of perceived levels of confidence in opioid prescribing by comparing them to actual practice patterns. Conducting a qualitative study will be beneficial in thoroughly understanding the viewpoints of physicians regarding their worries and the adverse occurrences experienced by patients.

## 5 Conclusion

Physicians possess a positive attitude and self-assurance when it comes to the use of opioids and the responsible management of opioid prescriptions. These can further be enhanced by implementing the criteria outlined in the Drug Abuse Control Master Plan of Pakistan regarding prescription opioids. Furthermore, there is a deficiency in interprofessional collaboration to address the issue of opioid use. This should be improved by implementing a stewardship program within the healthcare system, which would lead to better patient outcomes and help control the abuse of opioids. By updating medical, pharmacy, and nursing curricula and using opioid abuse risk assessment methods, we can reduce the increasing number of deaths caused by opioid overdose. We propose structured interprofessional education (IPE) between pharmacists and physicians during medical training. Pharmacists, with their specialized expertise in pharmacology and drug safety, could play a pivotal role in teaching medical students evidence-based opioid prescribing, risk mitigation (e.g., addiction screening and naloxone co-prescribing), and regulatory compliance. Pharmacists could be embedded in medical school curricula to co-teach opioid modules, emphasizing their unique “drug information authority” in balancing efficacy and safety. They could also participate in hospital rounds, modeling real-time collaboration (e.g., pain management planning and opioid tapering strategies) and reinforcing the value of interdisciplinary decision-making.

## Data Availability

The original contributions presented in the study are included in the article/Supplementary Material, further inquiries can be directed to the corresponding authors.
