# Supplementary material for: Evaluation of physicians’ opioid prescribing practices, attitudes, and interactions with other healthcare professionals in optimizing opioid prescribing in Pakistan
Source: Front Pharmacol. 2025 May 27;16:1491764. doi: 10.3389/fphar.2025.1491764 (PMC12149141; doi:10.3389/fphar.2025.1491764)
Supplement: Supplementary file 1 [file Supplementaryfile1.docx]

**Physician’s Opioids Prescribing Practices, Attitude and Interaction with Other Health Care Professionals in Managing Opioids Prescribing**

**Section 1: Demographic Information**

| 1. **Gender** | - Male - Female | **5. Age** | - <25 years - 25-35 years - 36-45 years - 46-55years - >56 years |
| --- | --- | --- | --- |
| 1. **Practice Experience** | - 1-5 years - 5-10 years - > 10 years | **6. Medical specialty** | - General practice - Obstetrics & Gynecology - Oncology - Surgery - Dental - Orthopedics - Others |
| 1. **Practice setting** | - Government - Private - Personal clinic - Both | **7. Nature of hospital care services** | - Primary care hospital - Secondary care hospital - Tertiary care hospital |
| 1. **Province** | - Punjab |  |  |

**Section 2: Knowledge about opioid stewardship**

1. Do you feel you have adequate knowledge about opioid stewardship?

| **No.** | **Questions** | **Responses** | |
| --- | --- | --- | --- |
|  |  | **Yes** | **No** |
|  | Do you know about opioids stewardship?  (Opioid stewardship refers to a series of strategies and interventions involving the appropriate procurement, storage, prescribing and use of opioids, as well as the disposal of unused opioids when opioids are appropriately prescribed for the treatment and management of specific medical conditions) |  |  |
|  | Do you know about opioids drugs? |  |  |
|  | Do you know about WHO analgesic ladder of pain management? |  |  |
|  | Do you know about the CDC Guideline for Prescribing Opioids for Chronic Pain? |  |  |
|  | Do you aware of the potential risks and adverse effects associated with opioid therapy? |  |  |
|  | Do you know opioid drugs are included in schedule G drugs by drug regulatory authority of Pakistan |  |  |
|  | Do you know opioid stewardship programs can help reduce the risk of opioid addiction and abuse? |  |  |
|  | Do you know about guidelines and recommendations for prescribing opioids in chronic pain management |  |  |
|  | Do you have adequate knowledge on the appropriate use of naloxone in opioid overdose situations? |  |  |
|  | Are you familiar with the risk factors for opioid abuse and addiction? |  |  |
|  | Do you aware of the resources available for patients who require assistance with opioid tapering or addiction treatment? |  |  |

**Section 3: prescribing practices and attitudes**

1. **In how many patients with non chronic pain have you prescribed opioids in past three months?**

- None
- 1-100
- 100-200
- >200
- Don’t know

1. **If no, why you don’t prescribe opioids**

- Preferred other analgesics therapy than opioids
- No patients consulted with chronic pain
- Uncertain about opioid selection in chronic pain
- Concern about opioid misuse and addiction

| **No.** | **Questions** | **Strongly Agree** | **Agree** | **Neutral** | **Disagree** | **Strongly Disagree** |
| --- | --- | --- | --- | --- | --- | --- |
|  | I am comfortable prescribing opioids for chronic pain. |  |  |  |  |  |
|  | I am confident in my clinical skills in prescribing opioids. |  |  |  |  |  |
|  | In my practice many pain patients experience substantial pain relief with opioids. |  |  |  |  |  |
|  | I find it satisfying to prescribe opioids to pain patients. |  |  |  |  |  |
|  | I am confident about incorporating non-opioid pain management strategies into my practice, such as physical therapy or cognitive-behavioral therapy |  |  |  |  |  |
|  | I am concerned about the potential for opioid diversion or abuse in my practice setting |  |  |  |  |  |
|  | I am confident in my ability to identify patients who may be at high risk for opioid abuse or addiction |  |  |  |  |  |

1. **How many of your patients have had an adverse event due to opioids in past six months?**

- None
- 1
- 2
- 3
- >4
- Don’t know

1. **Which opioid was involved in most recent adverse event? ____________________________________**
2. **Please select the factors that might have contributed to the adverse event: (can select multiple options)**

- Prescribed opioid dose was too high
- Patient didn’t contact the physician or pharmacist about symptoms
- Patient took more than prescribed opioids
- Patient took sedating drugs along with the opioid drug
- Patient injected, crushed or snorted the tablet
- Physician didn’t recognize seriousness of symptoms
- Patient refused to go to the emergency department
- Lack of communication between physician and patient
- Dispensing error
- Patient/caregiver misunderstanding about dosing
- Loss of tolerance following a period of non-compliance

1. **Is there any opioid adverse event reporting system in your practice setting?**

- Yes
- No

**Section 4:** **Physician’s concerns while prescribing opioids**

1. **Do your patients show concern, specifically with respect to their opioid prescription use?**

- Yes
- No
- Patient don’t aware of opioids drugs
- Don’t know

**Please rate the extent of your concerns while prescribing opioids**

| **No** | **Prescribing Concerns** | **Not at all**  **concerned** | **A little**  **concerned** | **Somewhat**  **concerned** | **Very**  **concerned** |
| --- | --- | --- | --- | --- | --- |
|  | Patient’s addiction to opioids |  |  |  |  |
|  | Non-compliance to therapy (e.g. missed appointments) |  |  |  |  |
|  | Trouble with legislation |  |  |  |  |
|  | Demand for early refill, unrealistic expectations of pain, demanding fit-in appointments, lost prescriptions |  |  |  |  |
|  | Overdose and abuse |  |  |  |  |
|  | Lack of resources to treat addiction |  |  |  |  |
|  | Patient’s unwillingness about opioids use |  |  |  |  |
|  | Lack of specialized pain clinics |  |  |  |  |

**Section 5**

**Interaction with other healthcare providers regarding opioids therapy (Pharmacist, Nurse)**

1. **How often do you interact with pharmacists regarding opioid therapy / prescriptions?**

- Weekly
- Monthly
- Quarterly
- Never

| **No.** | **Situations encountered with pharmacist regarding opioid therapy** | **Frequently** | **Sometimes** | **Not at all** |
| --- | --- | --- | --- | --- |
|  | Pharmacist was difficult to reach directly due to communication gap |  |  |  |
|  | Pharmacist called to verify something that is already stated on the Rx |  |  |  |
|  | Pharmacist requested a change in the wording of opioid prescriptions |  |  |  |
|  | Pharmacist challenged a prescription that I felt was appropriate |  |  |  |
|  | Pharmacist made a recommendation to a patient that I felt was inappropriate |  |  |  |
|  | Pharmacist interacted for minor issues or non-emergencies in opioid therapy management |  |  |  |
|  | Pharmacist dispensed opiate earlier than the time stated on the Rx e.g. on a weekly dispensed Rx |  |  |  |
|  | Pharmacist did not seem very knowledgeable about opioids |  |  |  |
|  | Pharmacist dispensed opioid medication without Rx when they could not reach the physician |  |  |  |
|  | Pharmacist did not adequately answer my concerns about opioids |  |  |  |

1. **How often do you interact with nurse regarding opioid therapy / prescriptions?**

- Weekly
- Monthly
- Quarterly
- Never

| **No.** | **Situations encountered with nurse regarding opioid therapy** | **Frequently** | **Sometimes** | **Not at all** |
| --- | --- | --- | --- | --- |
| **13** | Nurse felt uncomfortable while administering the opioids that I prescribed |  |  |  |
|  | Nurse showed reluctance with my opioid prescription to in patients |  |  |  |
|  | Nurse felt that the patient was drug seeking or difficult and I did not necessarily agree |  |  |  |
|  | Nurse pressured me to prescribe something to keep the patient calm or quiet e.g. benzodiazepines or other pain killers |  |  |  |
|  | Did not feel that nurse’s assessment of opioid intoxication, withdrawal or pain was accurate |  |  |  |

**Thanks for your cooperation**
